# Non-replication study of a genome-wide association study for hypertension and blood pressure in African Americans

**DOI:** 10.1186/1471-2350-13-27

**Published:** 2012-04-11

**Authors:** Srividya Kidambi, Soumitra Ghosh, Jane M Kotchen, Clarence E Grim, Shanthi Krishnaswami, Mary L Kaldunski, Allen W Cowley, Shailendra B Patel, Theodore A Kotchen

**Affiliations:** 1 Department of Medicine, Medical College of Wisconsin, Milwaukee, WI 53226, USA; 2 Division of Endocrinology, Clement J. Zablocki VA Medical Center, Milwaukee, WI 53295, USA; 3 Department of Pediatrics, Medical College of Wisconsin, Milwaukee, WI 53226, USA; 4 Institute for Health and Society, Medical College of Wisconsin, Milwaukee, WI 53226, USA; 5 Department of Physiology, Medical College of Wisconsin, Milwaukee, WI 53226, USA

## Abstract

**Background:**

A recent genome wide association study in 1017 African Americans identified several single nucleotide polymorphisms that reached genome-wide significance for systolic blood pressure. We attempted to replicate these findings in an independent sample of 2474 unrelated African Americans in the Milwaukee metropolitan area; 53% were women and 47% were hypertensives.

**Methods:**

We evaluated sixteen top associated SNPs from the above genome wide association study for hypertension as a binary trait or blood pressure as a continuous trait. In addition, we evaluated eight single nucleotide polymorphisms located in two genes (*STK-39 and CDH-13*) found to be associated with systolic and diastolic blood pressures by other genome wide association studies in European and Amish populations. TaqMan MGB-based chemistry with fluorescent probes was used for genotyping. We had an adequate sample size (80% power) to detect an effect size of 1.2-2.0 for all the single nucleotide polymorphisms for hypertension as a binary trait, and 1% variance in blood pressure as a continuous trait. Quantitative trait analyses were performed both by excluding and also by including subjects on anti-hypertensive therapy (after adjustments were made for anti-hypertensive medications).

**Results:**

For all 24 SNPs, no statistically significant differences were noted in the minor allele frequencies between cases and controls. One SNP (rs2146204) showed borderline association (p = 0.006) with hypertension status using recessive model and systolic blood pressure (p = 0.02), but was not significant after adjusting for multiple comparisons. In quantitative trait analyses, among normotensives only, rs12748299 was associated with SBP (p = 0.002). In addition, several nominally significant associations were noted with SBP and DBP among normotensives but none were statistically significant.

**Conclusions:**

This study highlights the importance of replication to confirm the validity of genome wide association study results.

## Background

Hypertension is a major contributor to the global disease burden with world-wide prevalence estimated to be ~ 26%, totaling ~ 1 billion people [1,2]. Currently, in the U.S., approximately 73 million Americans have hypertension, and the prevalence is particularly high in African Americans. Based on recent results of the National Health and Nutrition Examination Survey (NHANES), the age-adjusted hypertension prevalence is 39.1% in non-Hispanic blacks and 28.5% in non-Hispanic Whites [3]. Adoption, twin, and family studies document a significant heritable component to blood pressure levels and hypertension [4-6] and indicate that the heritability of blood pressure is in the range of 15-35% [7,8]. Hypertension before the age of 55 years occurs 3.8 times more frequently among persons with a positive family history of hypertension [9].

Genome-wide association studies (GWAS) are one strategy that is being exploited to identify the genetic contributions to hypertension. This strategy has been facilitated by the unraveling of the human genome, the HapMap project, and the availability of dense computer chips for genetic sequencing. In contrast to candidate gene approaches, GWAS offers the potential to identify novel mechanisms in the pathophysiology of hypertension. The first large GWAS for hypertension, performed by the Wellcome Trust Case Control Consortium (WTCCC) among British subjects (with 2000 cases and 3000 controls) in the [10], revealed no single nucleotide polymorphisms (SNPs) crossing the experimental threshold of statistical significance established at p < 10^-7^. Subsequently, several additional GWAS have been published, including 2 large-scale meta-analyses, demonstrating sporadic and inconsistent associations of SNPs with either blood pressure or hypertension [11-17]. Notably, these studies have been carried out in predominantly European and Amish populations.

In the first ever GWAS in African Americans (all subjects were residents of the Washington DC area), Adeyemo *et al *identified several SNPs reaching genome-wide significance for systolic blood pressure in or near genes: *PMS1, SLC24A4, YWHA7, IPO7, and CACANA1H *[18]. Two of these genes, *SLC24A4 *(a sodium/potassium/calcium exchanger) and *CACNA1H *(a voltage-dependent calcium channel), are potential candidate genes for blood pressure regulation and the latter is also a drug target for a class of calcium channel blockers. Some of the significant SNPs were replicated in a sample of West Africans [18]. Independent confirmation is necessary to validate these findings. Therefore, we investigated the association of SNPs identified by Adeyemo *et al *in an independent population of African American subjects from the mid-western United States. In addition, in these African American subjects, we also sought to replicate SNP variants recently identified by GWAS in European and Amish populations, and further confirmed in the study by Adeyemo *et al *[11,14,18].

## Results

A total of 2474 eligible subjects were genotyped of whom 53% (n = 1311) were women. Fifty-three percent (n = 1299) of the subjects were normotensives, and 47% (n = 1175) were hypertensives (Table 1). Of the hypertensives, 50% were on antihypertensive drug therapy. Hypertensives were older, had higher BMI, waist circumference, serum glucose, and cholesterol (p < 0.001) compared to normotensives. There were no significant differences in the gender composition or serum creatinine concentration.

**Table 1:** Demographic characteristics (mean ± SD) of cases and controls

Characteristic	All Hypertensives (n = 1175)	Untreated Hypertensives (n = 583)	Normotensives (n = 1299)	P-value between Normotensives and all Hypertensives
Age (years)	45 ± 7	45 ± 7	43 ± 7	< 0.0001

Female (%)	54	44	52	0.36

SBP (mmHg)	145 ± 20	149 ± 18	118 ± 10	

DBP (mmHg)	94 ± 12	98 ± 10	76 ± 8	

BMI (kg/m^2^)	29 ± 6	28 ± 6	27 ± 5	< 0.0001

Waist circumference (cm)	93 ± 13	90 ± 12	87 ± 11	< 0.0001

Serum glucose (mg/dL)	88 ± 17	87 ± 15	85 ± 15	< 0.0001

Serum creatinine (mg/dL)	0.90 ± 0.19	0.90 ± 0.19	0.9 ± 0.18	0.335

Serum cholesterol (mg/dL)	192 ± 43	191 ± 46	184 ± 38	< 0.0001

### Hardy-Weinberg Equilibrium

Genotype distributions were in Hardy-Weinberg equilibrium for all the SNPs except two (rs12757682 and rs1867226) when all the subjects were considered together as well as when normotensives were analyzed separately. Among hypertensives, in addition to rs12757682 and rs1867226, rs2146204 was also not in Hardy-Weinberg equilibrium.

### Case-control association analyses

Case-control association analyses were done separately for each SNP with hypertensives as cases and normotensives as controls using PLINK association analyses. We did not observe any statistically significant difference in the allele frequency or in the genotype distribution of the 24 SNPs between cases and controls (Additional file 1: Table S1). Genetic association analyses utilizing dominant and additive models did not reveal any significant associations between SNPs that were tested and hypertension status (Table 2- only additive model results are shown). One SNP (rs2146204) showed borderline association when recessive model was considered with a p-value of 0.006, but was not significant when adjusted for multiple comparisons and for age, gender, BMI and creatinine.

**Table 2:** Results of Case-control Genetic Association Analyses using Additive Model

SNP ID	Gene Symbol	Minor allele	MAF	p-value
rs9791170	P4HA2	T	0.431	0.86

rs12757682	(AC096631.2)	C	0.136	0.97

rs991316	ADH7	T	0.444	0.52

rs12748299	(AC096631.2)	G	0.124	0.93

rs1550576	ALDH1A2	T	0.224	0.44

rs7902529	(AL354747.12)	A	0.146	0.16

rs2146204*	(RP11-375 F2.1)	C	0.075	0.71

rs11160059	SLC24A4	T	0.067	0.16

rs17365948*	YWHAZ	T	0.012	0.43

rs5743185	PMS1	A	0.352	0.61

rs12279202*	IPO7	T	0.013	0.31

rs3751664*	CACNA1H	T	0.015	0.34

rs8039294	SV2B	G	0.451	0.44

rs10135446	NRXN3	T	0.145	0.21

rs9590141	ABCC4	A	0.107	0.76

rs1867226	PRC1	C	0.427	0.47

rs2063958*	STK 39	G	0.065	0.93

rs2390639	STK 39	G	0.337	0.24

rs11890527	STK 39	C	0.388	0.42

rs2203703	STK 39	C	0.415	0.36

rs11860907	CDH 13	C	0.471	0.43

rs7200009	CDH 13	T	0.098	0.74

rs16960421	CDH 13	T	0.164	0.94

rs17177428*	CDH 13	A	0.017	0.88

*p-values were obtained using modeling with Fishers exact test for dominant/recessive tests of association when the frequency of observations was < 5 per group

### Quantitative trait analyses

Quantitative trait associations were carried out using PLINK between these SNPs and systolic and diastolic blood pressure on subjects both with and without inclusion of treated hypertensive subjects (Additional file 1: Table S2). Blood pressures of treated hypertensives were adjusted for the effect of antihypertensive drug treatment before conducting the analyses. While this increased the sample size, the results were similar with both inclusion and exclusion of treated hypertensive subjects. Neither systolic nor diastolic blood pressures were significantly different among different genotypes of these 24 SNPs after statistical adjustment for multiple comparisons. However, without this statistical adjustment one SNP (rs2146204) that was previously associated with diastolic blood pressure in the report of Adeyemo *et al*, showed association with systolic blood pressure (p = 0.02).

In additional quantitative trait analyses, we evaluated normotensives and hypertensives separately (Additional file 1: Table S3). In normotensives only, SNP rs12748299 was associated with systolic blood pressure (p = 0.002). While this was statistically significant after adjustment for multiple comparisons, it was no longer significant after adjustment for age, sex, BMI, serum creatinine. Moreover, the association noted was with lower systolic blood pressure rather than previously associated hypertension status [18]. In addition, SNPs rs12757682 and rs17177428 showed borderline association with systolic and diastolic blood pressures (p = 0.04) respectively, but these were not significant after adjustment for multiple comparisons.

Quantitative trait analyses that included only hypertensive subjects (untreated and treated) showed no significant associations of blood pressure with any of the 24 SNPs after adjustment for multiple comparisons. However, the following several borderline associations were observed without statistical adjustment for multiple comparisons (Additional file 1: Table S3): a) SNP rs1550576 was associated with systolic blood pressure (p = 0.04); b) SNP rs17365948 was associated with systolic and diastolic blood pressure (p = 0.004 and 0.02 respectively); c) SNP rs7200009 is associated with systolic and diastolic blood pressure (p = 0.04); d) SNP rs17177428 was associated with systolic blood pressure (p = 0.04). The number of untreated hypertensive subjects was insufficient to permit quantitative trait analyses in this group of subjects.

### Regression analyses

Multiple linear regression analysis did not show a statistically significant impact of any of the alleles or genotypes on systolic and diastolic blood pressure in all subjects category (including treated subjects with stepped-up blood pressure), in all untreated subjects (normotensives plus untreated hypertensives), in normotensives alone or in hypertensives alone (Table 3). Independent variables included in the model were gender, age, body mass index, and serum creatinine. Multiple logistic regression analysis was also conducted with the same independent variables as linear regression with hypertension as a dependent binary trait. No significant impact of either genotypes or alleles of all 24 SNPs was noticed on the hypertension status.

**Table 3:** Results of Logistic and Linear Regression Analyses using minor allele as standard

SNP ID	HTN ß ± SE (p-value)	Untreated Subjects ß ± SE (p-value)	
		**SBP**	**DBP**

rs9791170	-0.052 ± 0.061 (0.39)	0.000 ± 0.005 (0.66)	0.000 ± 0.005 (0.96)

rs12757682	-0.068 ± 0.088 (0.44)	0.008 ± 0.007 (0.23)	0.008 ± 0.007 (0.82)

rs991316	-0.005 ± 0.061 (0.94)	0.000 ± 0.005 (0.44)	0.000 ± 0.005 (0.89)

rs12748299	-0.041 ± 0.091 (0.65)	0.007 ± 0.007 (0.33)	0.001 ± 0.007 (0.90)

rs1550576	0.037 ± 0.072 (0.60)	0.005 ± 0.006 (0.33)	0.011 ± 0.006 (0.06)

rs7902529	-0.087 ± 0.086 (0.31)	0.001 ± 0.006 (0.88)	0.004 ± 0.007 (0.54)

rs2146204*	-0.012 ± 0.117 (0.92)	0.000 ± 0.008 (0.81)	0.000 ± 0.009 (0.84)

rs11160059	0.140 ± 0.119 (0.24)	0.000 ± 0.009 (0.97)	-0.010 ± 0.010 (0.53)

rs17365948*	0.149 ± 0.281 (0.60)	0.000 ± 0.023 (0.85)	-0.020 ± 0.025 (0.53)

rs5743185	-0.065 ± 0.063 (0.30)	0.000 ± 0.005 (0.72)	0.000 ± 0.005 (0.55)

rs12279202*	-0.209 ± 0.270 (0.44)	-0.020 ± 0.021(0.35)	-0.010 ± 0.022 (0.69)

rs3751664*	-0.295 ± 0.253 (0.24)	-0.030 ± 0.020 (0.13)	-0.020 ± 0.021 (0.24)

rs8039294	0.029 ± 0.060 (0.63)	0.000 ± 0.005 (0.72)	0.001 ± 0.005 (0.81)

rs10135446	-0.123 ± 0.086 (0.15)	-0.010 ± 0.006 (0.04)	-0.010 ± 0.007 (0.16)

rs9590141	0.014 ± 0.097 (0.88)	0.003 ± 0.007 (0.66)	0.000 ± 0.008 (0.66)

rs1867226	-0.057 ± 0.061 (0.35)	0.005 ± 0.005 (0.27)	0.004 ± 0.005 (0.47)

rs2063958*	-0.022 ± 0.121(0.86)	-0.01 ± 0.009 (0.16)	-0.010 ± 0.010 (0.18)

rs2390639	-0.072 ± 0.064 (0.26)	-0.010 ± 0.005 (0.10)	-0.010 ± 0.005 (0.17)

rs11890527	-0.060 ± 0.062 (0.34)	0.000 ± 0.005 (0.32)	0.000 ± 0.005 (0.47)

rs2203703	-0.075 ± 0.061 (0.22)	0.000 ± 0.005 (0.36)	0.000 ± 0.005 (0.44)

rs11860907	-0.042 ± 0.061(0.22)	0.000 ± 0.005 (0.76)	0.000 ± 0.005 (0.75)

rs7200009	0.051 ± 0.103 (0.60)	0.003 ± 0.008 (0.73)	0.000 ± 0.009 (0.98)

rs16960421	-0.033 ± 0.082 (0.69)	0.003 ± 0.006 (0.60)	0.002 ± 0.007 (0.73)

rs17177428*	0.006 ± 0.234 (0.98)	0.020 ± 0.018 (0.26)	-0.010 ± 0.019 (0.53)

## Discussion

In this sample of African Americans, none of the SNPs evaluated were convincingly associated with hypertension as a binary trait or with blood pressure level as a quantitative trait. Thus, we were unable to confirm previously reported associations between *PMS1, SLC24A4, YWHA7, IPO7*, and *CACANA1H *with systolic and diastolic blood pressure. However, among normotensives only, one SNP (rs12748299) showed a significant association with systolic blood pressure even after adjusting for multiple comparisons, but was no longer significant after adjustment for age, sex, BMI and serum creatinine. This SNP was previously marginally associated with hypertension as a binary trait in the study by Adeyemo *et al*, but had not reached genome-wide significance in their study either. It is located on chromosome 1 in the intergenic region and its biological plausibility is unknown at this time.

We observed several suggestive, but not significant associations. SNP rs2146204, located on chromosome 1 in the intergenic region, was associated with systolic blood pressure in all subjects (treated and untreated). Similarly, rs12757682 which is also located on chromosome 1 in the intergenic region, showed borderline association with systolic blood pressure among normotensives. Among hypertensives, borderline associations (p > 0.002) were noted between rs1550576 (in the intergenic region near the gene *aldehyde dehydrogenase 1 family, member A2, ALDH1A2*) and systolic blood pressure, and rs17365948 (in the intronic region of the gene *tyrosine 3-monooxygenase/tryptophan 5-monooxygenase activation protein, zeta polypeptide, YWHAZ*) and systolic and diastolic blood pressure. The biological plausibility of these associations is currently unknown (Additional file 1: Table S4).

Similarly, Ehret *et al *attempted to replicate six top-associated SNPs (rs2820037 (1q43), rs6997709 (8q24), rs7961152 (12p12), rs11110912 (12q23), rs1937506 (13q21) and rs2398162 (15q26)) from WTCCC in the Family Blood Pressure Program (FBPP) cohort [10,19]. DNA was genotyped on 11,433 participants (39% African Americans, 39% European Americans, 21% Hispanic Americans) and the results showed that only one of these six SNPs (rs1937506 on 13q21) was *negatively *associated with systolic and diastolic blood pressure among European Americans. The same SNP showed a significant but opposite effect with systolic blood pressure in Hispanic Americans and was not associated in African Americans [19]. No replication could be shown for hypertension status. The associations that were closest to being suggestive could not be replicated, even in a well characterized sample such as the FBPP suggesting the difficulty in replicating the findings of GWAS [19]. The most significant criticism of the WTCCC was the use of young controls that could potentially become cases at a later age.

Inconsistent results among several small-scale GWAS [11-14,20] suggested that hypertension is polygenic disease with multiple low frequency genes acting in harmony to result in elevated blood pressure. To overcome some of these limiting factors, a large meta-analysis of CHARGE and BPgen Consortium results was conducted recently and several novel loci have been identified for blood pressure. Although these associations have not been replicated in independent populations, these results may be more robust than previous GWAS as the meta-analysis involved more than 60,000 subjects [15,16]. We did not choose to replicate these associations as these studies involved subjects primarily from European and Asian Indian ancestry.

On a similar note, a large GWAS for blood pressure among African Americans, the Candidate Gene Association Resource (CARe) Study, has recently been published. In a meta-analysis across five community-based cohorts, two novel loci were identified that reached statistical significance: rs2258119 on chromosome 21 with systolic blood pressure and rs10474346 on chromosome 5 with diastolic blood pressure [21]. However, neither of these associations were replicated in independent African American samples, again highlighting the difficulty in extending the findings of GWAS to independent populations. In addition, this study did not identify any of the loci identified by Adeyemo *et al*.

Even though the evidence for a genetic contribution remains robust, the effect of environment may be a major modifier, and this may be the reason for inconsistent data. Many common everyday activities can profoundly affect blood pressure, such as salt intake, exercise, stress, etc., and many of these are not quantitated in population-based studies. To minimize environmental and genetic variability, Wang *et al *carried out a GWAS of systolic and diastolic blood pressure in Amish subjects [11]. Strong association signals with several common variants in a serine/threonine kinase gene (*STK39) *were found and they confirmed these associations in an independent Amish and 4 non-Amish Caucasian samples [11]. Adeyemo *et al *found that several SNPs in this gene were associated with the blood pressure (9/136 for systolic blood pressure and 33/136 for diastolic blood pressure) in African Americans. Variants in *STK39 *may influence blood pressure by increasing *STK39 *expression and consequently altering renal sodium excretion [11]. We were unable to confirm the associations of 2 top-associated SNPs in this gene for systolic and diastolic blood pressure in our cohort despite having adequate power. These associations were not tested in CARe study by Fox *et al*.

Another novel locus contributing to blood pressure was identified by Org *et al *in a GWAS of a German population and is known as *Cadherin 13-Heart *(*CDH13*) [14]. *CDH13 *is a calcium-dependent cell adhesion glycoprotein and may mediate interaction between cells in heart. Several SNPs in this locus were also found to be associated with systolic and diastolic blood pressures among African Americans by Adeyemo *et al. *We tested the association of 4 of these SNPs with hypertension as a binary trait and blood pressure as a quantitative trait in our sample. We confirmed a borderline association (insignificant when considered with multiple tests) of rs17177428 and rs7200009 with blood pressure as quantitative trait among hypertensive and normotensive subjects. Again, this association was not tested in CARe study by Fox *et al*.

As evidenced by these studies, identification of genetic contributors to hypertension remains challenging for several reasons. Population heterogeneity, environmental effects, and sample size all contribute to these failures. Our study is also subject to these criticisms. In addition, as already eluded to, inheritance of blood pressure is polygenetic where in a single gene or combination of genes act in concert with above mentioned environmental exposures to contribute only a modest effect on blood pressure. Indeed, genetic variants identified by GWAS contribute to only a small effect on blood pressure (1-2 mmHg). Although this may have a population-based impact, this effect may not be discernible among individuals. Moreover, the difficulty of obtaining a standardized blood pressure phenotype cannot be over emphasized.

We also acknowledge the limitations of using commercially available tag SNPs. While it is possible to identify genetic variation with tag SNPs without genotyping every SNP in the chromosomal region and are widely used in genome-wide association studies, it may make replication of target SNPs difficult due to differences in linkage disequilibrium between two samples. For this reason, many studies have now adopted the technique of "local replication" in which SNPs surrounding tag SNP are also targeted, especially for SNPs that are not directly replicated from previous studies [22].

Apart from inherent difficulties in identifying genetic determinants of hypertension due to the phenotype itself as well as methods, we acknowledge that the differences between the populations investigated by our study and Adeyemo *et. al*. may account for non-replication of the GWAS. Since African Americans as an ethnic group are quite diverse with different origins, we recruited only the individuals whose parents were both born in the United States, with English as their first language, to achieve some uniformity in ethnicity. However, this may not have been sufficiently homogeneous population and the differences in the results may reflect different population admixture rates [23,24]. Since we do not know the admixture rate in our population, we were unable to adjust for that while Adeyemo et. al used genome-wide markers to compute principal components and used these in their models. These baseline differences in the African American population composition is also reflected in the allele frequencies of the various SNPs, e.g. CACNA1H (0.015 vs 0.109), IPO7 (0.013 vs 0.123), YWHAZ (0.012 vs 0.113), SLC24A4 (0.067 vs 0.178), PMS1 (0.352 vs 0.148) (Additional file 1: TableS 5).

There were several strengths to our study. Cases and controls were clearly defined with normotensives having blood pressures in the lower third of the population distribution of blood pressure (~ 3 SD below the untreated hypertensive means) to eliminate the bias of cases masquerading as controls or vice versa. Subjects with secondary hypertension were excluded including those with renal insufficiency. In addition, the mean age of the cases and controls are in mid-forties, which is sufficiently young to capture genetically determined blood pressure increases and old enough to capture most primary hypertensives. However, it is still possible some mis-classification of cases and controls might have occurred.

## Conclusions

While they are relatively easy to perform, interpretation of the results of successful association studies is neither straightforward nor always replicable. In addition, overestimation of genetic effect size in initial studies due to "winner's curse" may cause follow-up studies to be underpowered and so to fail [25]. Results of the current study and others do not replicate previously identified hypertension-related SNPs in GWAS. Non-genetic factors particularly environment, profoundly affect the impact of genes on blood pressure and hypertension and inherited phenotypes may potentially be a result of mechanisms without change in underlying DNA sequence. Consequently, in addition to focusing on replication of GWAS findings, future studies may need to move beyond DNA-based sequence approaches and may involve the evaluation of heritable changes in gene expression [26].

## Methods

### Subjects and measurements

The study was approved by the Froedtert Memorial Lutheran Hospital/Medical College of Wisconsin Institutional Review Board. Unrelated African Americans subjects between the ages of 18-55 years were recruited from a variety of community resources and health care providers within the Milwaukee area. Subjects were defined as African Americans based on self-identification, birth in the continental United States, both parents reported as being African Americans, and English as the native language. All subjects were evaluated during a single outpatient visit and were considered to have hypertension if standardized outpatient measurement of systolic blood pressure was ≥ 140 mmHg, diastolic blood pressure was ≥ 90 mmHg and/or if they were taking antihypertensive medications. Blood pressures of normotensives were in the lower third of the distribution of population blood pressures. Pregnant subjects, subjects with diabetes mellitus (fasting blood glucose ≥ 126 mg/dL (6.93 mmol/L) or random blood sugar > 200 mg/dL (11 mmol/L), and subjects with serum creatinine concentrations ≥ 1.3 mg/dL (114.92 μmol/L) were excluded.

After subjects provided informed consent, standardized measurements of blood pressure and anthropometric measurements including height, weight, and waist circumference were acquired. Blood pressures were taken by trained and certified personnel according to the American Heart Association guidelines by the Shared Care Method using a sphygmomanometer [27]. After being seated for at least 5 minutes, 2 readings were obtained from each arm using an appropriate sized arm cuff and the average of the arm with the higher measurement was used for the visit reading. Waist circumference was taken at the narrowest point between the umbilicus and superior iliac crest. Serum glucose was measured with an automated glucose oxidase enzymatic assay. Plasma cholesterol was measured using an enzymatic procedure.

### Selection of SNPs and genotyping

SNPs associated with hypertension and blood pressure in the genome wide association study by Adeyemo *et al *among AAs were selected for the study [18]. As shown in Additional file 1: Table S4, these include the following: a) seven top-associated SNPs for hypertension as a binary trait b) five top associated SNPs for systolic blood pressure as a continuous trait and c) four top-associated SNPs for diastolic blood pressure. In addition, 8 variants in 2 genes (*STK39 and CHD13*) identified in European and Amish populations [11,14] and replicated by Adeyemo *et al *[18] in African Americans were also selected for replication in the current study. Among these SNPs, only a few had reached genome-wide significance for systolic BP in the study by Adeyemo et. al. There are the SNPs in or near the genes: PMS1 (rs5743185), SLC24A4 (rs11160059), YWHA7 (rs17365948), IPO7 (rs12279202), and CACANA1H (rs3751664). In addition, STK39 and CHD 13 gene variants reached genome wide significance in non-African American populations.

DNA was extracted manually from peripheral white blood cell pellets using reagents from Qiagen (Qiagen Inc. Valencia, CA, USA) and precipitated using isopropanol [28]. TaqMan MGB-based chemistry (Applied Biosystems, Foster City, CA, USA) with fluorescent probes was used for all genotyping. Genotypes were called using the manufacturer's software and all data were inspected manually. In our laboratory, this method has ~99% concordance between duplicate samples, and produces a genotype for 97.4% of all samples attempted with 95% validity. Approximately 1.8% of the samples were deemed indeterminate or failures.

### Statistical methods

Continuous variables were reported as means ± standard deviation (SD). Differences in the distributions of all selected phenotypes between hypertensive and normotensive subjects were determined either by Student's t-test or by Wilcoxon rank sum test, depending upon the distribution of variables. Both SAS software version 9.1 (SAS Institute, CARY, NC) and PLINK [29] were used for analyses. PLINK was used to perform genetic association analyses considering log-additive, dominant, and recessive models for both quantitative and qualitative traits. Deviations of the observed allele frequencies from those expected under Hardy-Weinberg equilibrium were tested by the χ^2 ^-test. In case-control analyses, odds ratios for hypertension were calculated using the major allele as reference. When analyzing systolic and diastolic blood pressures as continuous traits, to account for the treatment effect amongst those on anti-hypertensive medications, we used stepped addition of blood pressures to treated subjects based on number of drugs a subject was taking. As previously described by others [30], stepped increments of 8/4 mmHg, 14/10 mmHg, and 20/16 mmHg were added to measured systolic and diastolic blood pressures of treated subjects taking 1, 2, and ≥ 3 drug classes, respectively. These stepped increments were chosen to achieve an average increase of 10/5 mmHg in treated subjects. Analyses for blood pressure as a continuous trait were also performed excluding subjects on antihypertensive medications.

P-values < 0.002 were deemed as significant after adjustment for multiple tests. One-way ANOVA was conducted to assess if there was a difference in the blood pressure among all 3 genotypes for each SNP. Multiple linear regression analyses were carried out with systolic and diastolic blood pressures as continuous dependent variables and multiple logistic regression with hypertension as categorical dependent variable. SNPs were represented as categorical variables either as minor allele or as homozygous genotype of minor allele in both logistic regression and linear regression analyses. Other independent variables included gender, age, BMI, and creatinine in the same model.

*Power calculations *were performed using the program Quanto v1.1 (James Gauderman, University of Southern California, USA) [31]. Power for unmatched case-control (case-control ratio = 1:1) studies was estimated using actual allelic frequencies of tested genetic markers ranging from 0.01 to 0.5, a log-additive model, a disease prevalence of 33% and a type 1 error rate of 0.002. Sample sizes were estimated for genetic effect sizes (odds ratios) of 1.3 to 2.3 with the log-additive model. Due to different minor allele frequencies (MAFs) for different SNPs, we listed the minimal effect size (odds ratio) that could be detected with the available sample size for the qualitative trait analyses (Additional file 1: Table S6). To estimate power in a continuous trait analysis, systolic and diastolic blood pressures were used as a continuous outcome for independent subjects, with an additive model and a type 1 error rate of 0.002 and an effect size of 1% variance. A sample size of 781 was needed to detect a difference of 1 mmHg of blood pressure.

## Abbreviations

NHANES: National health and nutrition examination survey; GWAS: Genome wide association study; SNP: Single nucleotide polymorphisms; WTCCC: Wellcome trust case control consortium; PMS1: Post-meiotic segregation increased 1; IPO7: Importin 7; CACANA1H: Calcium channel voltage-dependent: T type: alpha 1H subunit; BMI: Body mass index; ALDH1A2: Aldehyde dehydrogenase 1 family member A2; YWHAZ: Tyrosine 3-monooxygenase/tryptophan 5-monooxygenase activation protein zeta polypeptide; FBPP: Family blood pressure program; DNA: Deoxyribonucleic acid; STK 39: Serine threonine kinase 39; CDH13: Cadherin 13- heart; CHARGE: Cohorts for heart and aging research in genomic epidemiology; Global BPgen: Global blood pressure genetics; CARe: Candidate gene association resource; P4HA2: Prolyl 4-hydroxylase alpha polypeptide II; ADH 7: Alcohol dehydrogenase 7 (class IV) mu or sigma polypeptide; HTN: Hypertension; Chr: Chromosome; rs: reference SNP number; SLC24A4: Solute carrier family 24 (Na/Ca/K exchanger) member 4; SV2B: synaptic vesicle glycoprotein 2B; NRXN3: Neurexin3; ABCC4: ATP-binding cassette (ABC) sub-family C (CFTR/MRP): member 4; PRC1: Protein regulator of cytokinesis 1; SBP: Systolic blood pressure; DBP: Diastolic blood pressure; MAF: Minor allele frequency.

## Competing interests

The authors declare that they have no competing interests.

## Authors' contributions

SK participated in design the study, data acquisition, interpret and analyze the data and drafted the manuscript. SG helped interpret the data, JK participated in design and data acquisition, CG participated in design and data acquisition, ShK helped data analyses and manuscript writing, MK assisted in data acquisition and review of the manuscript, AC participated in design of the study, SP participated in design, analyses and interpretation of the data and drafting manuscript, TK participated in design, data acquisition, interpretation and data analyses and drafted the manuscript. All authors read and approved the final manuscript.

## Pre-publication history

The pre-publication history for this paper can be accessed here:

http://www.biomedcentral.com/1471-2350/13/27/prepub

## Supplementary Material

Additional file 1 **Table S1**. Genotype distribution of all 24 SNPs in cases and controls. Table S2 - Quantitative trait analyses. Table S3- Quantitative trait analyses in normotensive and hypertensive subjects. Table S4 - List of single-nucleotide polymorphisms (SNPs) genotyped. Table S5- Results of current study in comparison to study by Adeyemo et. al. Table S6 - Power calculation of effect size detectable based on minor allele frequency in case-control analyses. Click here for file

## References

[B1] Chobanian AV Bakris GL Black HR Cushman WC Green LA Izzo JL Jr Jones DW Materson BJ Oparil S Wright JT Jr Seventh Report of the Joint National Committee on Prevention, Detection, Evaluation, and Treatment of High Blood Pressure Hypertension 2003 42 1206 1252 10.1161/01.HYP.0000107251.49515.c2 14656957

[B2] Kearney PM Whelton M Reynolds K Muntner P Whelton PK He J Global burden of hypertension: analysis of worldwide data Lancet 2005 365 217 223 15652604 10.1016/S0140-6736(05)17741-1

[B3] Egan BM Zhao Y Axon RN US trends in prevalence, awareness, treatment, and control of hypertension, 1988-2008 JAMA 2010 303 2043 2050 10.1001/jama.2010.650 20501926

[B4] Hunt SC Hasstedt SJ Kuida H Stults BM Hopkins PN Williams RR Genetic heritability and common environmental components of resting and stressed blood pressures, lipids, and body mass index in Utah pedigrees and twins Am J Epidemiol 1989 129 625 638 2916556 10.1093/oxfordjournals.aje.a115175

[B5] Williams PD Puddey IB Martin NG Beilin LJ Genetic and environmental covariance of serum cholesterol and blood pressure in female twins Atherosclerosis 1993 100 19 31 10.1016/0021-9150(93)90064-2 8318060

[B6] Somes GW Harshfield GA Alpert BS Goble MM Schieken RM Genetic influences on ambulatory blood pressure patterns. The Medical College of Virginia Twin Study Am J Hypertens 1995 8 474 478 10.1016/0895-7061(95)00017-J 7662223

[B7] Biron P Mongeau JG Bertrand D Familial aggregation of blood pressure in 558 adopted children Can Med Assoc J 1976 115 773 774 974967 PMC1878814

[B8] Williams RR Hunt SC Hasstedt SJ Hopkins PN Wu LL Berry TD Stults BM Barlow GK Schumacher MC Lifton RP Are there interactions and relations between genetic and environmental factors predisposing to high blood pressure? Hypertension 1991 18 I29 I37 1889856 10.1161/01.hyp.18.3_suppl.i29

[B9] Hunt SC Williams RR Barlow GK A comparison of positive family history definitions for defining risk of future disease J Chronic Dis 1986 39 809 821 10.1016/0021-9681(86)90083-4 3760109

[B10] Genome-wide association study of 14,000 cases of seven common diseases and 3,000 shared controls Nature 2007 447 661 678 10.1038/nature05911 17554300 PMC2719288

[B11] Wang Y O'Connell JR McArdle PF Wade JB Dorff SE Shah SJ Shi X Pan L Rampersaud E Shen H From the Cover: Whole-genome association study identifies STK39 as a hypertension susceptibility gene Proc Natl Acad Sci USA 2009 106 226 231 10.1073/pnas.0808358106 19114657 PMC2629209

[B12] Saxena R Voight BF Lyssenko V Burtt NP de Bakker PI Chen H Roix JJ Kathiresan S Hirschhorn JN Daly MJ Genome-wide association analysis identifies loci for type 2 diabetes and triglyceride levels Science 2007 316 1331 1336 17463246 10.1126/science.1142358

[B13] Sabatti C Service SK Hartikainen AL Pouta A Ripatti S Brodsky J Jones CG Zaitlen NA Varilo T Kaakinen M Genome-wide association analysis of metabolic traits in a birth cohort from a founder population Nat Genet 2009 41 35 46 10.1038/ng.271 19060910 PMC2687077

[B14] Org E Eyheramendy S Juhanson P Gieger C Lichtner P Klopp N Veldre G Doring A Viigimaa M Sober S Genome-wide scan identifies CDH13 as a novel susceptibility locus contributing to blood pressure determination in two European populations Hum Mol Genet 2009 18 2288 2296 10.1093/hmg/ddp135 19304780 PMC2685752

[B15] Newton-Cheh C Johnson T Gateva V Tobin MD Bochud M Coin L Najjar SS Zhao JH Heath SC Eyheramendy S Genome-wide association study identifies eight loci associated with blood pressure Nat Genet 2009 41 666 676 10.1038/ng.361 19430483 PMC2891673

[B16] Levy D Ehret GB Rice K Verwoert GC Launer LJ Dehghan A Glazer NL Morrison AC Johnson AD Aspelund T Genome-wide association study of blood pressure and hypertension Nat Genet 2009 41 677 687 10.1038/ng.384 19430479 PMC2998712

[B17] Cheng LSC Davis RC Raffel LJ Xiang AH Wang N Quinones M Wen PZ Toscano E Diaz J Pressman S Coincident Linkage of Fasting Plasma Insulin and Blood Pressure to Chromosome 7q in Hypertensive Hispanic Families Circulation 2001 104 1255 1260 10.1161/hc3601.096729 11551876

[B18] Adeyemo A Gerry N Chen G Herbert A Doumatey A Huang H Zhou J Lashley K Chen Y Christman M Rotimi C A genome-wide association study of hypertension and blood pressure in African Americans PLoS Genet 2009 5 e1000564 10.1371/journal.pgen.1000564 19609347 PMC2702100

[B19] Ehret GB Morrison AC O'Connor AA Grove ML Baird L Schwander K Weder A Cooper RS Rao DC Hunt SC Replication of the Wellcome Trust genome-wide association study of essential hypertension: the Family Blood Pressure Program Eur J Hum Genet 2008 16 1507 1511 10.1038/ejhg.2008.102 18523456 PMC2585612

[B20] Cho YS Go MJ Kim YJ Heo JY Oh JH Ban HJ Yoon D Lee MH Kim DJ Park M A large-scale genome-wide association study of Asian populations uncovers genetic factors influencing eight quantitative traits Nat Genet 2009 41 527 534 10.1038/ng.357 19396169

[B21] Fox ER Young JH Li Y Dreisbach AW Keating BJ Musani SK Liu K Morrison AC Ganesh S Kutlar A Association of genetic variation with systolic and diastolic blood pressure among African Americans: the Candidate Gene Association Resource study Hum Mol Genet 2011 20 2273 2284 10.1093/hmg/ddr092 21378095 PMC3090190

[B22] Validation in genetic association studies Briefings in Bioinformatics 2011 12 253 258 10.1093/bib/bbq074 21546448

[B23] Patterson N Hattangadi N Lane B Lohmueller KE Hafler DA Oksenberg JR Hauser SL Smith MW O'Brien SJ Altshuler D Methods for high-density admixture mapping of disease genes Am J Hum Genet 2004 74 979 1000 10.1086/420871 15088269 PMC1181990

[B24] Smith MW Patterson N Lautenberger JA Truelove AL McDonald GJ Waliszewska A Kessing BD Malasky MJ Scafe C Le E A high-density admixture map for disease gene discovery in african americans Am J Hum Genet 2004 74 1001 1013 10.1086/420856 15088270 PMC1181963

[B25] Xiao R Boehnke M Quantifying and correcting for the winner's curse in genetic association studies Genet Epidemiol 2009 33 453 462 10.1002/gepi.20398 19140131 PMC2706290

[B26] Wang X Snieder H Genome-Wide Association Studies and Beyond: What's Next in Blood Pressure Genetics? Hypertension 2010 56 1035 1037 10.1161/HYPERTENSIONAHA.110.157214 21060002 PMC4102918

[B27] Grim CM Grim CE A curriculum for the training and certification of blood pressure measurement for health care providers Can J Cardiol 1995 11 Suppl H 38H 42H 7489543

[B28] Bogner PN KA Songalis T, CaGJ WB Extraction of Nucleic acids Molecular diagnostics for the clinical laboratorian 2006 2 New Jersey: Humana Press 25 30

[B29] Purcell S Neale B Todd-Brown K Thomas L Ferreira MA Bender D Maller J Sklar P de Bakker PI Daly MJ Sham PC PLINK: a tool set for whole-genome association and population-based linkage analyses Am J Hum Genet 2007 81 559 575 10.1086/519795 17701901 PMC1950838

[B30] Cui JS Hopper JL Harrap SB Antihypertensive treatments obscure familial contributions to blood pressure variation Hypertension 2003 41 207 210 10.1161/01.HYP.0000044938.94050.E3 12574083

[B31] Gauderman WJMJ Quanto 1.1 Book Quanto 1.1 (Editor ed.^eds.) pp. A computer program for power and sample size calculations for genetic-epidemiology studies City; 2006:A computer program for power and sample size calculations for genetic-epidemiology studies

