# Supplementary material for: Non-replication study of a genome-wide association study for hypertension and blood pressure in African Americans
Source: BMC Med Genet. 2012 Apr 11;13:27. doi: 10.1186/1471-2350-13-27 (PMC3349540; doi:10.1186/1471-2350-13-27)
Supplement: Additional file 1 — Table S1. Genotype distribution of all 24 SNPs in cases and controls. Table S2 - Quantitative trait analyses. Table S3- Quantitative trait analyses in normotensive and hypertensive subjects. Table S4 - List of single-nucleotide polymorphisms (SNPs) genotyped. Table S5- Results of current study in comparison to study by Adeyemo et. al. Table S6 - Power calculation of effect size detectable based on minor allele frequency in case-control analyses. [file 1471-2350-13-27-S1.DOCX]

**Supplementary Table 1. Genotype distribution of all 24 SNPs in cases and controls.**

| SNPs/ Locus/  Closest gene | Previously  associated trait | MAF in all  subjects | Genotype/  Allele N (%) | Cases  (n=1175) | Controls  (n=1299) | *Odds Ratio*  *(p-value)** |
| --- | --- | --- | --- | --- | --- | --- |
| rs9791170  Chr 5  P4HA2 | HTN | 0.431 (T) | CC | 379 | 424 |  |
|  |  |  | TT | 214 | 248 |  |
|  |  |  | CT | 573 | 618 |  |
|  |  |  | MAF | 0.429 | 0.432 | 0.99 (0.86) |
| rs12757682  Chr 1 | HTN | 0.136 (C) ‡ | TT | 885 | 985 |  |
|  |  |  | CC | 42 | 51 |  |
|  |  |  | CT | 229 | 246 |  |
|  |  |  | MAF | 0.135 ‡ | 0.136‡ | 1.00 (0.97) |
| rs991316  Chr 4  ADH7 | HTN | 0.444 (T) | CC | 361 | 379 |  |
|  |  |  | TT | 222 | 249 |  |
|  |  |  | CT | 563 | 635 |  |
|  |  |  | MAF | 0.439 | 0.449 | 0.96 (0.52) |
| rs12748299  Chr 1  AC096631.2 | HTN | 0.124 (G) | CC | 892 | 983 |  |
|  |  |  | GG | 18 | 19 |  |
|  |  |  | CG | 252 | 277 |  |
|  |  |  | MAF | 0.124 | 0.123 | 1.01 (0.93) |
| rs1550576  Chr 15  ALDH1A2 | HTN | 0.224 (T) | CC | 702 | 785 |  |
|  |  |  | TT | 70 | 65 |  |
|  |  |  | CT | 395 | 435 |  |
|  |  |  | MAF | 0.229 | 0.220 | 1.06 (0.43) |
| rs11160059  Chr 14  SLC24A4 | SBP | 0.067 (T) | CC | 1001 | 1132 |  |
|  |  |  | TT | 5 | 6 |  |
|  |  |  | CT | 159 | 149 |  |
|  |  |  | MAF | 0.073 | 0.063 | 1.17 (0.16) |
| rs17365948  Chr 8  YWHAZ | SBP | 0.012 (T) | CC | 1133 | 1261 |  |
|  |  |  | TT | 0 | 0 |  |
|  |  |  | CT | 30 | 27 |  |
|  |  |  | MAF | 0.013 | 0.010 | 1.23 (0.43) |
| rs8039294  Chr 15 | SBP | 0.451 (G) | TT | 334 | 398 |  |
|  |  |  | GG | 234 | 259 |  |
|  |  |  | GT | 592 | 628 |  |
|  |  |  | MAF (%) | 0.457 | 0.446 | 1.05 (0.44) |
| rs10135446  Chr 14  NRXN3 | DBP | 0.145 (T) | GG | 855 | 921 |  |
|  |  |  | TT | 23 | 32 |  |
|  |  |  | GT | 273 | 322 |  |
|  |  |  | MAF (%) | 0.139 | 0.151 | 0.90 (0.21) |
| rs9590141  Chr 13  ABCC4 | DBP | 0.107 (A) | GG | 925 | 1034 |  |
|  |  |  | AA | 13 | 17 |  |
|  |  |  | AG | 225 | 237 |  |
|  |  |  | MAF | 0.108 | 0.105 | 1.03 (0.76) |
| rs1867226  Chr 15  PRC1 | DBP | 0.427 (C) ‡ | GG | 413 | 449 |  |
|  |  |  | CC | 231 | 275 |  |
|  |  |  | CG | 516 | 559 |  |
|  |  |  | MAF | 0.422‡ | 0.432 ‡ | 0.96 (0.45) |
| rs3751664  Chr 16  CACNA1H | SBP | 0.015 (T) | CC | 1135 | 1248 |  |
|  |  |  | TT | 1 | 1 |  |
|  |  |  | CT | 29 | 41 |  |
|  |  |  | MAF | 0.013 | 0.017 | 0.80 (0.33) |
| rs2063958  Chr 2  STK39-1 | SBP | 0.065 (G) | AA | 1008 | 1126 |  |
|  |  |  | GG | 4 | 10 |  |
|  |  |  | AG | 143 | 146 |  |
|  |  |  | MAF | 0.065 | 0.065 | 1.01 (0.93) |
| rs2390639  Chr 2  STK39-2 | SBP | 0.337 (G) | AA | 520 | 538 |  |
|  |  |  | GG | 126 | 143 |  |
|  |  |  | AG | 505 | 592 |  |
|  |  |  | MAF | 0.329 | 0.345 | 0.93 (0.24) |
| rs11890527  Chr 2  STK-39-3 | DBP | 0.388 (C) | TT | 430 | 465 |  |
|  |  |  | CC | 157 | 193 |  |
|  |  |  | CT | 570 | 617 |  |
|  |  |  | MAF | 0.382 | 0.393 | 0.95 (0.42) |
| rs2203703  Chr 2  STK-39-4 | DBP | 0.415 (C) | CC | 393 | 419 |  |
|  |  |  | TT | 182 | 218 |  |
|  |  |  | CT | 579 | 639 |  |
|  |  |  | MAF | 0.409 | 0.421 | 0.95 (0.37) |
| rs11860907  Chr 16  CDH13-1 | SBP | 0.471 (C) | AA | 330 | 349 |  |
|  |  |  | CC | 249 | 288 |  |
|  |  |  | AC | 575 | 644 |  |
|  |  |  | MAF | 0.465 | 0.476 | 0.96 (0.43) |
| rs7200009  Chr 16  CDH13-2 | SBP | 0.098 (T) | CC | 901 | 1011 |  |
|  |  |  | TT | 12 | 13 |  |
|  |  |  | CT | 197 | 213 |  |
|  |  |  | MAF | 0.100 | 0.097 | 1.03 (0.73) |
| rs16960421  Chr 16  CDH13-3 | DBP | 0.164 (T) | GG | 802 | 892 |  |
|  |  |  | TT | 31 | 33 |  |
|  |  |  | GT | 314 | 350 |  |
|  |  |  | MAF | 0.164 | 0.163 | 1.01 (0.94) |
| rs17177428  Chr 16  CDH13-4 | DBP | 0.017 (A) | GG | 1103 | 1229 |  |
|  |  |  | AA | 0 | 0 |  |
|  |  |  | AG | 39 | 42 |  |
|  |  |  | MAF | 0.017 | 0.017 | 1.03 (0.88) |
| rs5743185  Chr 2  PMS1 | SBP | 0.352 (A) | GG | 498 | 541 |  |
|  |  |  | AA | 149 | 172 |  |
|  |  |  | AG | 500 | 560 |  |
|  |  |  | MAF | 0.348 | 0.355 | 0.97 (0.60) |
| rs12279202  Chr 11  IPO7 | SBP | 0.013 (T) | CC | 1126 | 1234 |  |
|  |  |  | TT | 0 | 0 |  |
|  |  |  | CT | 26 | 37 |  |
|  |  |  | MAF | 0.011 | 0.015 | 0.77 (0.32) |
| rs7902529  Chr 10  AL354747.12 | HTN | 0.146 (A) | TT | 840 | 905 |  |
|  |  |  | AA | 24 | 36 |  |
|  |  |  | AT | 263 | 310 |  |
|  |  |  | MAF | 0.138 | 0.153 | 0.89 (0.15) |
| rs2146204  Chr 1  RP11-375F2.1 | HTN | 0.075 (C) | AA | 960 | 1050 |  |
|  |  |  | CC | 14 | 3 |  |
|  |  |  | AC | 136 | 184 |  |
|  |  |  | MAF | 0.074‡ | 0.077 | 0.96 (0.70) |

*P-value for differences in allelic frequency

‡Not in Hardy-Weinberg equilibrium

HTN: Hypertension SBP: Systolic blood pressure DBP: Diastolic blood pressure Chr: Chromosome

**Supplementary Table 2. Quantitative trait analyses**

| SNPs/ Locus/  Closest gene | Previously  Associated trait | Genotype/  Allele | All Subjects (n= 2474) | | Normotensive plus untreated hypertensive subjects (n=1882) | |
| --- | --- | --- | --- | --- | --- | --- |
|  |  |  | Systolic Blood Pressure* | Diastolic Blood Pressure* | Systolic Blood Pressure* | Diastolic Blood Pressure* |
| rs9791170  Chr 5  P4HA2 | HTN | CC | 133 ± 22 | 86 ± 15 | 128 ± 20 | 83 ± 14 |
|  |  | TT | 133 ± 22 | 87 ± 14 | 128 ± 20 | 83 ± 13 |
|  |  | CT | 133 ± 22 | 86 ± 14 | 128 ± 19 | 83 ± 13 |
|  |  | p-value | 0.68 | 0.60 | 1.00 | 0.93 |
| rs12757682  Chr 1 | HTN | TT | 133 ± 22 | 86 ± 14 | 127 ± 19 | 83 ± 13 |
|  |  | CC | 133 ± 22 | 86 ± 14 | 128 ± 20 | 82 ± 12 |
|  |  | CT | 134 ± 21 | 87 ± 15 | 129 ± 20 | 83 ± 14 |
|  |  | p-value | 0.37 | 0.77 | 0.20 | 0.81 |
| rs991316  Chr 4  ADH7 | HTN | CC | 134 ± 23 | 87± 15 | 128 ± 20 | 83 ± 13 |
|  |  | TT | 133 ± 23 | 86 ± 15 | 127 ± 20 | 83 ± 14 |
|  |  | CT | 133 ± 21 | 86 ± 14 | 128 ± 18 | 83 ± 13 |
|  |  | p-value | 0.52 | 0.68 | 0.62 | 0.94 |
| rs12748299  Chr 1  AC096631.2 | HTN | CC | 133 ± 22 | 86 ± 14 | 127 ± 19 | 83 ± 13 |
|  |  | GG | 132 ± 24 | 88 ± 16 | 123 ± 17 | 84 ± 13 |
|  |  | CG | 134 ± 21 | 87 ± 15 | 130 ± 20 | 83 ± 13 |
|  |  | p-value | 0.31 | 0.73 | 0.06 | 0.88 |
| rs1550576  Chr 15  ALDH1A2 | HTN | CC | 133 ± 22 | 87± 14 | 127 ± 19 | 82 ± 13 |
|  |  | TT | 132 ± 20 | 86 ± 13 | 127 ± 19 | 84 ± 13 |
|  |  | CT | 134 ± 22 | 87 ± 14 | 129 ± 19 | 84 ± 13 |
|  |  | p-value | 0.45 | 0.55 | 0.22 | 0.14 |
| rs11160059  Chr 4  SLC24A4 | SBP | CC | 133 ± 22 | 86 ± 15 | 128 ± 20 | 83 ± 13 |
|  |  | TT | 135 ± 16 | 87 ± 15 | 131 ± 18 | 82 ± 13 |
|  |  | CT | 133 ± 20 | 86 ± 14 | 127 ± 17 | 82 ± 13 |
|  |  | p-value | 0.92 | 0.87 | 0.87 | 0.82 |
| rs17365948  Chr 8  YWHAZ | SBP | CC | 133 ± 22 | 86 ± 14 | 128 ± 19 | 83 ± 13 |
|  |  | TT |  |  |  |  |
|  |  | CT | 139 ± 28 | 90 ± 17 | 129 ± 26 | 82 ± 14 |
|  |  | p-value | 0.07 | 0.13 | 0.95 | 0.61 |
| rs8039294  Chr 15 | SBP | TT | 133 ± 22 | 86 ± 14 | 128 ± 19 | 83 ± 13 |
|  |  | GG | 133 ± 21 | 86 ± 14 | 127 ± 18 | 83 ± 12 |
|  |  | GT | 134 ± 22 | 86 ± 15 | 128 ± 20 | 83 ± 13 |
|  |  | p-value | 0.42 | 0.77 | 0.30 | 0.95 |
| rs10135446  Chr 14  NRXN3 | DBP | GG | 133 ± 22 | 87± 15 | 128 ± 19 | 83 ± 13 |
|  |  | TT | 130 ± 23 | 84 ± 16 | 126 ± 22 | 80 ± 14 |
|  |  | GT | 133 ± 23 | 87 ± 15 | 126 ± 19 | 83 ± 13 |
|  |  | p-value | 0.51 | 0.46 | 0.14 | 0.23 |
| rs9590141  Chr 13  ABCC4 | DBP | GG | 133 ± 22 | 86 ± 14 | 127 ± 19 | 83 ± 13 |
|  |  | AA | 130 ± 21 | 83 ± 13 | 128 ± 22 | 81 ±14 |
|  |  | AG | 134 ± 23 | 86 ± 15 | 129 ± 19 | 83 ± 14 |
|  |  | p-value | 0.63 | 0.54 | 0.56 | 0.85 |
| rs1867226  Chr 15  PRC1 | DBP | GG | 133 ± 22 | 86 ± 15 | 127 ± 19 | 83 ± 13 |
|  |  | CC | 133 ± 23 | 86 ± 15 | 129 ± 20 | 83 ± 13 |
|  |  | CG | 133 ± 22 | 86 ± 14 | 128 ± 19 | 83 ± 13 |
|  |  | p-value | 0.99 | 0.99 | 0.58 | 0.71 |
| rs3751664  Chr 16  CACNA1H | SBP | CC | 133 ± 22 | 86 ± 15 | 128 ± 19 | 83 ± 13 |
|  |  | TT | 134 ± 21 | 100 ± 25 | 119 ± 0 | 82 ± 0 |
|  |  | CT | 131 ± 20 | 85 ± 12 | 124 ± 17 | 81 ± 11 |
|  |  | p-value | 0.73 | 0.36 | 0.43 | 0.71 |
| rs2063958  Chr 2  STK39-1 | SBP | AA | 133 ± 26 | 86± 14 | 128 ± 19 | 83 ± 13 |
|  |  | GG | 125 ± 17 | 80 ± 14 | 122 ± 15 | 77 ± 10 |
|  |  | AG | 134 ± 23 | 87 ± 15 | 127 ± 19 | 83 ± 13 |
|  |  | p-value | 0.30 | 0.17 | 0.46 | 0.24 |
| rs2390639  Chr 2  STK39-2 | SBP | AA | 134 ± 22 | 87± 14 | 129 ± 20 | 84 ± 13 |
|  |  | GG | 134 ± 23 | 86 ± 15 | 128 ± 20 | 83 ± 13 |
|  |  | AG | 133 ± 22 | 86 ± 14 | 127 ± 18 | 82 ± 13 |
|  |  | p-value | 0.62 | 0.32 | 0.12 | 0.07 |
| rs11890527  Chr 2  STK-39-3 | DBP | TT | 133 ± 21 | 86 ± 15 | 128 ± 20 | 83 ± 13 |
|  |  | CC | 133 ± 22 | 86 ± 15 | 127 ± 20 | 83 ± 13 |
|  |  | CT | 134 ± 23 | 86 ± 15 | 128 ± 19 | 83 ± 13 |
|  |  | p-value | 0.67 | 0.32 | 0.65 | 0.55 |
| rs2203703  Chr 2  STK-39-4 | DBP | CC | 133 ± 22 | 86 ± 14 | 128 ± 20 | 83 ± 13 |
|  |  | TT | 133 ± 23 | 86 ± 15 | 127 ± 19 | 83 ± 13 |
|  |  | CT | 133 ± 22 | 86 ± 15 | 128 ± 19 | 83 ± 13 |
|  |  | p-value | 0.76 | 0.98 | 0.63 | 0.66 |
| rs11860907  Chr 16  CDH13-1 | SBP | AA | 133 ± 21 | 86 ± 14 | 128 ± 19 | 83 ± 12 |
|  |  | CC | 133 ± 23 | 86 ± 15 | 127 ± 20 | 83 ± 14 |
|  |  | AC | 133 ± 23 | 86 ± 15 | 128 ± 19 | 83 ± 13 |
|  |  | p-value | 0.96 | 1.00 | 0.69 | 0.86 |
| rs7200009  Chr 16  CDH13-2 | SBP | CC | 133 ± 23 | 86 ± 14 | 128 ± 20 | 83 ± 13 |
|  |  | TT | 136 ± 26 | 90 ± 19 | 128 ± 20 | 84 ± 14 |
|  |  | CT | 133 ± 19 | 86 ± 14 | 128 ± 18 | 83 ± 13 |
|  |  | p-value | 0.89 | 0.51 | 0.81 | 0.94 |
| rs16960421  Chr 16  CDH13-3 | DBP | GG | 133 ± 22 | 86 ± 15 | 128 ± 19 | 83 ± 13 |
|  |  | TT | 137 ± 26 | 87 ± 15 | 129 ± 19 | 82 ± 13 |
|  |  | GT | 134 ± 22 | 86 ± 14 | 128 ± 19 | 83 ± 13 |
|  |  | p-value | 0.29 | 0.87 | 0.63 | 0.57 |
| rs17177428  Chr 16  CDH13-4 | DBP | GG | 133 ± 22 | 86 ± 14 | 128 ± 19 | 83 ± 13 |
|  |  | AA |  |  |  |  |
|  |  | AG | 136 ± 23 | 85 ± 15 | 131 ± 23 | 82 ± 14 |
|  |  | p-value | 0.31 | 0.47 | 0.20 | 0.64 |
| rs5743185  Chr 2  PMS1 | SBP | GG | 133 ± 22 | 86± 15 | 128 ± 19 | 83 ± 13 |
|  |  | AA | 134 ± 23 | 86 ± 15 | 128 ± 19 | 83 ± 13 |
|  |  | AG | 133 ± 22 | 86 ± 15 | 128 ± 20 | 83 ± 13 |
|  |  | p-value | 0.73 | 0.96 | 0.98 | 0.90 |
| rs12279202  Chr 11  IPO7 | SBP | CC | 133 ± 22 | 86 ± 15 | 128 ± 19 | 83 ± 13 |
|  |  | TT |  |  |  |  |
|  |  | CT | 130 ± 22 | 84 ± 14 | 125 ± 20 | 81 ± 11 |
|  |  | p-value | 0.22 | 0.25 | 0.23 | 0.45 |
| rs7902529  Chr 10  AL354747.12 | HTN | TT | 133 ± 22 | 86 ± 15 | 128 ± 19 | 83 ± 13 |
|  |  | AA | 130 ± 21 | 85 ± 14 | 128 ± 22 | 84 ± 14 |
|  |  | AT | 133 ± 21 | 86 ± 14 | 128 ± 19 | 83 ± 13 |
|  |  | p-value | 0.41 | 0.75 | 1.00 | 0.85 |
| rs2146204  Chr 1  RP11-375F2.1 | HTN | AA | 133 ± 22 | 86 ± 14 | 128 ± 20 | 82 ± 13 |
|  |  | CC | 145 ± 22 | 91 ± 18 | 140 ± 13 | 90 ± 13 |
|  |  | AC | 131 ± 22 | 85 ± 14 | 126 ± 19 | 82 ± 13 |
|  |  | p-value | **0.02** | 0.09 | **0.03** | 0.10 |

*Mean ± SD mm Hg

**Supplementary Table 3. Quantitative trait analyses in normotensive and hypertensive subjects.**

| SNPs/ Locus/  Closest gene | Previously associated  trait | Genotype/  Allele | Normotensive subjects  (n=1299) | | Hypertensive subjects  (n=1175) | |
| --- | --- | --- | --- | --- | --- | --- |
|  |  |  | Systolic Blood Pressure* | Diastolic Blood Pressure* | Systolic Blood Pressure* | Diastolic Blood Pressure* |
| rs9791170  Chr 5  P4HA2 | HTN | CC | 118 ± 10 | 76 ± 8 | 149 ± 21 | 97± 13 |
|  |  | TT | 118 ± 10 | 77 ± 7 | 150 ± 19 | 98 ± 12 |
|  |  | CT | 118 ± 10 | 76 ± 8 | 150 ± 20 | 97 ± 12 |
|  |  | p-value | 0.94 | 0.30 | 0.58 | 0.44 |
| rs12757682  Chr 1 | HTN | TT | 118 ± 10 | 76 ± 8 | 150 ± 20 | 97± 12 |
|  |  | CC | 119 ± 10 | 77 ± 8 | 151 ± 20 | 97 ± 12 |
|  |  | CT | 119 ± 9 | 77 ± 8 | 150 ± 19 | 98 ± 13 |
|  |  | p-value | **0.04** | 0.70 | 0.89 | 0.97 |
| rs991316  Chr 4  ADH7 | HTN | CC | 118 ± 10 | 76 ± 7 | 149 ± 19 | 97± 12 |
|  |  | TT | 118 ± 10 | 76 ± 7 | 150 ± 20 | 97 ± 13 |
|  |  | CT | 118 ± 10 | 76 ± 8 | 151 ± 21 | 98 ± 12 |
|  |  | p-value | 0.53 | 0.98 | 0.37 | 0.93 |
| rs12748299  Chr 1  AC096631.2 | HTN | CC | 118 ± 10 | 76 ± 7 | 150 ± 20 | 97± 12 |
|  |  | GG | 114 ± 10 | 76 ± 10 | 151 ± 19 | 100 ± 10 |
|  |  | CG | 120 ± 9 | 77 ± 8 | 150 ± 19 | 97 ± 13 |
|  |  | p-value | **0.002** | 0.59 | 0.86 | 0.54 |
| rs1550576  Chr 15  ALDH1A2 | HTN | CC | 118 ± 10 | 76 ± 8 | 150 ± 20 | 97± 12 |
|  |  | TT | 118 ± 8 | 78 ± 6 | 144 ± 20 | 94 ± 12 |
|  |  | CT | 119 ± 9 | 77 ± 8 | 150 ± 19 | 98 ± 11 |
|  |  | p-value | 0.08 | 0.21 | **0.04** | 0.11 |
| rs11160059  Chr 4  SLC24A4 | SBP | CC | 118 ± 10 | 76 ± 7 | 150 ± 20 | 98 ± 12 |
|  |  | TT | 123 ± 8 | 76 ± 8 | 149 ± 9 | 99 ± 12 |
|  |  | CT | 118 ± 10 | 76 ± 8 | 146 ± 17 | 96 ± 11 |
|  |  | p-value | 0.52 | 0.22 | 0.05 | 0.14 |
| rs17365948  Chr 8  YWHAZ | SBP | CC | 118 ± 10 | 76 ± 8 | 149 ± 20 | 97± 12 |
|  |  | TT |  |  |  |  |
|  |  | CT | 116 ± 10 | 75 ± 9 | 160 ± 21 | 102 ± 13 |
|  |  | p-value | 0.19 | 0.48 | **0.004** | **0.02** |
| rs8039294  Chr 15 | SBP | TT | 118 ± 10 | 76 ± 7 | 150 ± 20 | 97± 12 |
|  |  | GG | 118 ± 10 | 77 ± 8 | 148 ± 19 | 97 ± 12 |
|  |  | GT | 118 ± 10 | 76 ± 7 | 150 ± 20 | 97 ± 12 |
|  |  | p-value | 0.96 | 0.38 | 0.58 | 0.94 |
| rs10135446  Chr 14  NRXN3 | DBP | GG | 118 ±10 | 76 ± 8 | 149 ± 20 | 97± 12 |
|  |  | TT | 116 ± 9 | 73 ± 7 | 151 ± 21 | 100 ± 13 |
|  |  | GT | 118 ± 9 | 77 ± 7 | 151 ± 20 | 98 ± 12 |
|  |  | p-value | 0.20 | 0.08 | 0.52 | 0.13 |
| rs9590141  Chr 13  ABCC4 | DBP | GG | 118 ±10 | 77 ± 8 | 151 ± 20 | 98± 12 |
|  |  | AA | 117 ± 11 | 75 ± 8 | 147 ± 18 | 94 ± 10 |
|  |  | AG | 118 ± 11 | 75 ± 7 | 150 ± 20 | 97 ± 12 |
|  |  | p-value | 0.70 | 0.10 | 0.71 | 0.41 |
| rs1867226  Chr 15  PRC1 | DBP | GG | 118 ± 10 | 76 ± 7 | 150 ± 20 | 97± 12 |
|  |  | CC | 118 ± 9 | 76 ± 7 | 151 ± 21 | 98 ± 12 |
|  |  | CG | 118 ± 10 | 76 ± 8 | 149 ± 19 | 97 ± 12 |
|  |  | p-value | 0.74 | 0.89 | 0.55 | 0.54 |
| rs3751664  Chr 16  CACNA1H | SBP | CC | 118 ± 10 | 76 ± 8 | 150 ± 20 | 97± 12 |
|  |  | TT | 119 ± 1 | 82 ± 0 | 149 ± 0 | 118 ± 0 |
|  |  | CT | 118 ± 8 | 77 ± 7 | 149 ± 18 | 96 ± 9 |
|  |  | p-value | 0.97 | 0.63 | 0.97 | 0.24 |
| rs2063958  Chr 2  STK39-1 | SBP | AA | 118 ± 9 | 76 ± 7 | 150 ± 20 | 97± 12 |
|  |  | GG | 118 ± 10 | 76 ± 7 | 145 ± 13 | 97 ± 12 |
|  |  | AG | 118 ± 10 | 76 ± 8 | 151 ± 21 | 98 ± 13 |
|  |  | p-value | 0.86 | 0.56 | 0.62 | 0.66 |
| rs2390639  Chr 2  STK39-2 | SBP | AA | 118 ± 9 | 77 ± 7 | 150 ± 20 | 97± 12 |
|  |  | GG | 118 ± 10 | 76 ± 7 | 152 ± 20 | 98 ± 13 |
|  |  | AG | 118 ± 10 | 76 ± 8 | 150 ± 19 | 97 ± 12 |
|  |  | p-value | 0.77 | 0.58 | 0.52 | 0.90 |
| rs11890527  Chr 2  STK-39-3 | DBP | TT | 118 ± 9 | 77 ± 7 | 149 ± 19 | 97± 12 |
|  |  | CC | 118 ± 10 | 76 ± 7 | 151 ± 19 | 98 ± 12 |
|  |  | CT | 118 ± 9 | 76 ± 8 | 150 ± 21 | 98 ± 12 |
|  |  | p-value | 0.86 | 0.51 | 0.51 | 0.28 |
| rs2203703  Chr 2  STK-39-4 | DBP | CC | 118 ± 9 | 77 ± 7 | 149 ± 20 | 96± 12 |
|  |  | TT | 118 ± 10 | 76 ± 7 | 151 ± 20 | 98 ± 12 |
|  |  | CT | 118 ± 10 | 76 ± 8 | 150 ± 20 | 98 ± 12 |
|  |  | p-value | 0.51 | 0.59 | 0.32 | 0.14 |
| rs11860907  Chr 16  CDH13-1 | SBP | AA | 119 ± 10 | 76 ± 7 | 148 ± 19 | 96± 12 |
|  |  | CC | 118 ± 10 | 76 ± 8 | 151 ± 21 | 98 ± 12 |
|  |  | AC | 118 ± 10 | 76 ± 8 | 150 ± 20 | 98 ± 13 |
|  |  | p-value | 0.57 | 0.76 | 0.29 | 0.17 |
| rs7200009  Chr 16  CDH13-2 | SBP | CC | 118 ± 10 | 76 ± 8 | 150 ± 21 | 98± 12 |
|  |  | TT | 115 ± 11 | 77 ± 9 | 158 ± 19 | 104 ± 16 |
|  |  | CT | 119 ± 9 | 76 ± 7 | 147± 17 | 96 ± 12 |
|  |  | p-value | 0.09 | 0.90 | **0.04** | **0.04** |
| rs16960421  Chr 16  CDH13-3 | DBP | GG | 118 ± 10 | 76 ± 7 | 149 ± 19 | 97± 12 |
|  |  | TT | 121 ± 12 | 78 ± 9 | 154 ± 25 | 97 ± 13 |
|  |  | GT | 119 ± 10 | 77 ± 8 | 150 ± 20 | 97 ± 12 |
|  |  | p-value | 0.09 | 0.24 | 0.46 | 0.84 |
| rs17177428  Chr 16  CDH13-4 | DBP | GG | 118 ± 10 | 76 ± 8 | 150 ± 20 | 97± 12 |
|  |  | AA |  |  |  |  |
|  |  | AG | 117 ± 8 | 74 ± 7 | 156 ± 17 | 97 ± 12 |
|  |  | p-value | 0.51 | **0.04** | **0.04** | 0.86 |
| rs5743185  Chr 2  PMS1 | SBP | GG | 118 ± 10 | 76 ± 8 | 149 ± 20 | 98 ± 11 |
|  |  | AA | 119 ± 10 | 76 ± 8 | 152 ± 21 | 98 ± 12 |
|  |  | AG | 118 ± 10 | 77 ± 7 | 150 ± 20 | 97 ± 13 |
|  |  | p-value | 0.55 | 0.46 | 0.44 | 0.54 |
| rs12279202  Chr 11  IPO7 | SBP | CC | 118 ± 10 | 76 ± 8 | 150 ± 20 | 97± 12 |
|  |  | TT |  |  |  |  |
|  |  | CT | 118 ±10 | 77 ± 7 | 147 ± 22 | 94 ± 15 |
|  |  | p-value | 0.91 | 0.55 | 0.39 | 0.15 |
| rs7902529  Chr 10  AL354747.12 | HTN | TT | 118 ± 10 | 76 ± 8 | 150 ± 20 | 97± 12 |
|  |  | AA | 120 ± 10 | 77 ± 7 | 145 ± 24 | 97 ± 13 |
|  |  | AT | 118 ± 9 | 77 ± 7 | 149 ± 19 | 97 ± 11 |
|  |  | p-value | 0.50 | 0.39 | 0.38 | 0.82 |
| rs2146204  Chr 1  RP11-375F2.1 | HTN | AA | 118 ± 10 | 76 ± 7 | 150 ± 20 | 97± 12 |
|  |  | CC | 122 ± 4 | 73 ± 2 | 150 ± 22 | 97 ± 12 |
|  |  | AC | 118 ± 9 | 76 ± 8 | 149 ± 21 | 97 ± 12 |
|  |  | p-value | 0.76 | 0.73 | 0.88 | 0.46 |

*Mean ± SD mm Hg

HTN: Hypertension SBP: Systolic blood pressure DBP: Diastolic blood pressure Chr: Chromosome

**Supplementary Table 4: Results of current study in comparison to study by Adeyemo et. al.**

|  |  |  | **Current study** | | **Adeyemo et. al** | |
| --- | --- | --- | --- | --- | --- | --- |
| **SNP ID** | **Gene Symbol** | **Minor allele** | **MAF** | **p-value** | **MAF** | **p-value** |
| rs9791170 | P4HA2 | T | 0.431 | 0.85 | 0.434 | 5.10 E-07 |
| rs12757682 | (AC096631.2) | C | 0.136 | 0.87 | 0.132 | 2.59 E-05 |
| rs991316 | ADH7 | T | 0.444 | 0.43 | 0.451 | 3.45 E-06 |
| rs12748299 | (AC096631.2) | G | 0.124 | 0.96 | 0.132 | 1.66 E-05 |
| rs1550576 | ALDH1A2 | T | 0.224 | 0.64 | 0.142 | 1.03 E-05 |
| rs7902529 | (AL354747.12) | A | 0.146 | 0.23 | 0.141 | 6.14 E-06 |
| rs2146204 | (RP11-375F2.1) | C | 0.075 | 0.29 | 0.087 | 2.97 E-06 |
| rs11160059 | SLC24A4 | T | 0.067 | 0.13 | 0.178 | 1.54 E-08 |
| rs17365948 | YWHAZ | T | 0.012 | 0.50 | 0.113 | 1.59 E-08 |
| rs5743185 | PMS1 | A | 0.352 | 0.65 | 0.148 | 2.09 E-11 |
| rs12279202 | IPO7 | T | 0.013 | 0.37 | 0.123 | 4.80 E-08 |
| rs3751664 | CACNA1H | T | 0.015 | 0.34 | 0.109 | 6.71 E-08 |
| rs8039294 | SV2B | G | 0.451 | 0.24 | 0.483 | 7.73 E-06 |
| rs10135446 | NRXN3 | T | 0.145 | 0.26 | 0.1298 | 4.47 E-06 |
| rs9590141 | ABCC4 | A | 0.107 | 0.65 | 0.122 | 8.76 E-07 |
| rs1867226 | PRC1 | C | 0.427 | 0.75 | 0.464 | 5.80 E-07 |
| rs2063958 | STK 39 | G | 0.065 | 0.71 | - | 0.01 |
| rs2390639 | STK 39 | G | 0.337 | 0.15 | - | 0.012 |
| rs11890527 | STK 39 | C | 0.388 | 0.72 | - | 1.02 x 10^-4^ |
| rs2203703 | STK 39 | C | 0.415 | 0.52 | - | 2.17 x 10^-4^ |
| rs11860907 | CDH 13 | C | 0.471 | 0.46 | - | 5.71 x 10^-4^ |
| rs7200009 | CDH 13 | T | 0.098 | 0.73 | - | 1.08 x 10^-3^ |
| rs16960421 | CDH 13 | T | 0.164 | 0.98 | - | 1.82 x 10^-3^ |
| rs17177428 | CDH 13 | A | 0.017 | 0.91 | - | 3.42 x 10^-3^ |

**Supplementary Table 5. List of single-nucleotide polymorphisms (SNPs) genotyped**

| SNP ID | Associated Gene Name | SNP Location | | Associated  Trait | Known Gene Function |
| --- | --- | --- | --- | --- | --- |
| rs9791170 | Prolyl 4-hydroxylase, alpha polypeptide II (P4HA2) | Chr 5/ Intergenic | | HTN | A key enzyme in collagen synthesis and catalyzes the formation of 4-hydroxyproline. |
| rs12757682 | (AC096631.2) | Chr 1 | | HTN |  |
| rs991316 | Alcohol dehydrogenase 7 (class IV), mu or sigma polypeptide (ADH7) | Chr 4/ Intergenic | | HTN | Involved in retinol oxidation. |
| rs12748299 | (AC096631.2) | Chr 1/ Intergenic | | HTN |  |
| rs1550576 | Aldehyde dehydrogenase 1 family, member A2 (ALDH1A2) | Chr 15/ Intergenic | | HTN | An enzyme involved in synthesis of retinoic acid, a signaling molecule that functions in developing and adult tissues. |
| rs7902529 | (AL354747.12) | Chr 10/ Intergenic | | HTN |  |
| rs2146204 | (RP11-375F2.1) | Chr 1/ Intergenic | | HTN |  |
| rs11160059 | Solute carrier family 24 (Na/Ca/K exchanger), member 4 (SLC24A4) | Chr 14/ Intronic | | SBP | Transports intracellular Ca and K ion in exchange for extracellular Na ions. |
| rs17365948 | Tyrosine 3-monooxygenase/ tryptophan 5-monooxygenase activation protein, zeta polypeptide (YWHAZ) | Chr 8/  Intronic | | SBP | Mediates signal transduction by binding to phosphoserine- proteins e.g. IRS1, suggesting a role in insulin sensitivity. |
| rs5743185 | Post-meiotic segregation increased 1 (S. cerevisiae) (PMS 1) | Chr 2/ Intronic | | SBP | Involved in the repair of DNA mismatches, and mutations in this gene cause hereditary non-polyposis colorectal cancer type 3. |
| rs12279202 | Importin 7 (IPO7) | Chr 11/Intronic | | SBP | Importin complex mediates nuclear import of proteins with nuclear localization signal. |
| rs3751664 | Calcium channel, voltage-dependent, T type, alpha 1H subunit (CACNA1H) | Chr 16/Non- synonymous coding | | SBP | A protein in the voltage-dependent Ca channel, mutations in this gene lead to childhood absence epilepsy. |
| rs8039294 | synaptic vesicle glycoprotein 2B (SV2B) | Chr15/ Intronic | | DBP | In rats, it mediates the uptake of neuro-transmitters into vesicles. |
| rs10135446 | Neurexin3 (NRXN3) | Chr 14/ Intergenic | | DBP | Function as cell adhesion molecules and receptors in the nervous system. |
| rs9590141 | ATP-binding cassette (ABC), sub-family C (CFTR/MRP), member 4 (ABCC4) | Chr13/ Intergenic | | DBP | Function of ABCC4 is unknown, may play role in cell-detoxification by pumping organic anions. |
| rs1867226 | Protein regulator of cytokinesis 1  (PRC 1) | Chr 15/ Intronic | | DBP | Involved in cytokinesis, and has been shown to be a substrate of several cyclin-dependent kinases. |
| Serine threonine kinase 39 | | | | | |
| rs2063958 | Serine threonine kinase 39-1  (STK 39-1) | | Chr 2/ Intronic | SBP | This enzyme is thought to function in cellular stress response pathway, particularly hypotonic stress. |
| rs2390639 | Serine threonine kinase 39-1  (STK 39-2) | | Chr 2/ Intronic | SBP |  |
| rs11890527 | Serine threonine kinase 39-3  (STK 39-3) | | Chr 2/ Intronic | DBP |  |
| rs2203703 | Serine threonine kinase 39-4  (STK 39-4) | | Chr 2/ Intronic | DBP |  |
| Cadherin 13 (Heart) | | | | | |
| rs11860907 | Cadherin 13 – Heart -1  (CDH 13-1) | | Chr 16/ Intronic | SBP | Ca-dependent cell-cell adhesion glycoprotein and is a mediator of cell-cell interaction in the heart and may act as a negative regulator of neural cell growth. |
| rs7200009 | Cadherin 13 – Heart -2  (CDH 13-2) | | Chr 16/ Intronic | SBP |  |
| rs16960421 | Cadherin 13 – Heart -3  (CDH 13-3) | | Chr 16/ Intronic | DBP |  |
| rs17177428 | Cadherin 13 – Heart -4  (CDH 13-4) | | Chr 16/ Intronic | DBP |  |

HTN: Hypertension Chr: Chromosome SBP: Systolic blood pressure DBP: Diastolic blood pressure

**Supplementary Table 6. Power calculation of effect size detectable based on minor allele frequency in case-control analyses**

| MAF | SNP ID | Effect size detected for a p <0.05 | Effect size detected for a p <0.002 |
| --- | --- | --- | --- |
| 0.25 – 0.50 | rs2390639, rs5743185, rs11890527, rs2203703, rs1867226, rs9791170, rs991316, rs8039294, rs11860907 | 1.2 | 1.3 |
| 0.13 – 0.24 | rs7902529, rs12757682, rs10135446, rs16960421, rs1550576, | 1.3 | 1.4 |
| 0.11-0.12 | rs9590141, rs12748299 | 1.3 | 1.5 |
| 0.09-0.10 | rs7200009, | 1.4 | 1.5 |
| 0.06 – 0.08 | rs2146204, rs11160059, rs2063958 | 1.4 | 1.6 |
| 0.04 – 0.05 | None | 1.5 | 1.7-1.8 |
| 0.03 | None | 1.8 | 2.0 |
| 0.02 | rs3751664, rs17177428 | 1.8 | 2.3 |
| 0.01 | rs17365948, rs12279202 | 2.0 | 3.1 |

MAF: Minor Allele Frequency
